# Pre-Administration of Blackberry Extracts in Induced Ischemia Reperfusion Events in Rodents

**DOI:** 10.3390/metabo13111114

**Published:** 2023-10-28

**Authors:** Asahi Oda, Yoji Hakamata, Eiji Kobayashi

**Affiliations:** Division of Basic Science, School of Veterinary Nursing and Technology, Nippon Veterinary and Life Science University, 1-7-1 Kyonancho, Musashino-shi, Tokyo 180-8602, Japan

**Keywords:** blackberry extracts, nanoparticles, ischemia reperfusion, pre-administration, gerbil

## Abstract

Blackberries are abundant in substances that have antioxidative and other effects, and technologies for enhancing the effectiveness of their incorporation into the body are being developed. The effectiveness of such substances has been investigated in various models, including rodent ischemia models. While a test substance can be administered either before or after an event, healthy foods are generally pre-administered prophylactically in experiments. Pre-administration may have the potential to elevate the blood concentration of the active substance sufficiently prior to the event and/or induce adaptive changes in the ischemic tolerance of the recipient through long-term pre-administration. Based on the recently reported 2-week pre-administration of blackberries in a rat model, we investigated the pre-administration of blackberry extracts in a hyperlipidemia model using Mongolian gerbils. We then discussed the effects of the pre-administration on the treated animals before an ischemic event.

Blackberries are known to contain flavonols, phenolic acids, ellagic acid, vitamin C, and vitamin E, producing high levels of anthocyanins. Anthocyanins have been reported to possess significant scavenging properties against various reactive oxygen species such as superoxide radicals, hydrogen peroxide, and hydroxyl radicals, offering protective effects against excessive stress in brain neurons [1] and pancreatic islets [2]. However, the effectiveness of specific components within blackberry fruits and leaves varies depending on the harvesting period [3] and the extraction method used [4,5]. Additionally, relying solely on extracts may not always yield the desired effects, prompting explorations into the application of nanotechnology using metal nanoparticles such as silver nanoparticles (AgNPs) [6].

Recently, intriguing research has been carried out on the effects and mechanisms of AgNP-loaded blackberry leaf extracts (BBE) in a rat model of ischemia reperfusion injury (IRI) [7]. The IRI model involved male Wistar rats in which liver ischemia was induced by clamping the portal area for 30 min. Two-week pre-treatment with AgNP-loaded BBE effectively prevented liver damage caused by ischemia reperfusion. Comprehensive virtual screening and molecular dynamics simulations suggested that cyanidin and pelargonidin glucoside, the primary annotated anthocyanins, may act as PLA2 inhibitors [7].

Healthy foods widely consumed by people and with no or very few adverse effects are attracting attention of researchers investigating the human equivalent dose. Rodent models have been used to investigate their mechanism. Our research focused on the pre-administration of BBE. In their experimental protocol, previous researchers administered the investigated BBE varieties to the rats 14 d prior to the induction of ischemia reperfusion. Pre-administration is contemplated as a practice in which the administration of an active substance begins several days prior to the event with the intended effects of achieving a sufficient increase in the blood concentration of the active substance before the event and inducing a transformation in the recipient’s ischemic tolerance through prolonged pre-administration. In this study, we focused on preventive therapy before the occurrence of events such as ischemia. Specifically, we used ischemia/reperfusion gerbil models [8], which are a subset of rats prone to hyperlipidemia. Under normal dietary conditions, gerbils exhibit triglyceride (TG) levels exceeding 300 mg/dL. On a daily basis, we orally administered compressed blackberry juice extracted from blackberries (Kobayashi Regeneration Institute LLC, Wakayama) at a dose of 1.5 mL per 100 g of body weight (BW) for 14 consecutive days (N = 4). In detail, a predetermined volume of BBE (1.5 mL/100 g) per BW was administered into the stomach using a gastric tube at specified times (once in the morning and evening each). Daily food intake, changes in BW, and blood concentrations of total cholesterol, TG, and high-density lipoprotein (HDL) were measured. The control group received an equivalent amount of distilled water using the same procedure (N=3). This experiment was conducted with the approval of the University’s Animal Ethics Committee (Experimental Permission No. 2022S-16).

Our results indicated that the daily food intake did not differ between the blackberry-treated and vehicle (distilled water) groups. Furthermore, no significant difference was noted in BW changes between the two groups. However, the 14-day pre-administration of BBE led to a reduction in triglyceride levels and an increase in HDL cholesterol levels in gerbils. Specifically, on the 14th day, the blackberry-treated group exhibited TG and HDL concentrations of 3388.0 ± 1522.5 mg/dL and 32.4 ± 9.2 mg/dL, respectively, whereas the vehicle group showed concentrations of 4191.0 ± 705.6 mg/dL and 24.2 ± 14.7 mg/dL, respectively.

We wondered if the observed changes in blood metabolism reported by Fathi et al. following pre-administration of BBE [7] are involved in alleviating hepatic ischemia reperfusion injury. It is intriguing to explore whether differences occur in the effects of short-term pre-administration and long-term administration. We believe that preventative administration of BBE as a health supplement is an important possible strategy for the older adults with a risk of ischemia disease.

## Data Availability

Raw data were generated at Nippon Veterinary and Life Science University. Derived data supporting the findings of this study are available from the author Y.H. upon reasonable request. Data are not publicly available due to privacy.
